# Analysis of risk factors for pneumothorax after particle implantation in the treatment of advanced lung cancer after surgery and establishment of a nomogram prediction model

**DOI:** 10.3389/fmed.2024.1428456

**Published:** 2024-10-16

**Authors:** Tingting Ding, Shanhu Hao, Zhiguo Wang, Wenwen Zhang, Guoxu Zhang

**Affiliations:** Northern Theater Command General Hospital, Shenyang, China

**Keywords:** particle implantation, close-range radiotherapy, pneumothorax, Nomogram model, advanced lung cancer

## Abstract

**Objective:**

To analyze the risk factors for pneumothorax after particle implantation in the treatment of advanced lung cancer and to construct and validate a nomogram prediction model.

**Methods:**

A retrospective analysis was conducted on 148 patients who underwent ^125^I particle implantation for advanced lung cancer at the *** from December 2022 to December 2023. Potential risk factors were identified using univariate logistic regression analysis, followed by a multivariate logistic regression analysis to evaluate the predictive factors for pneumothorax. Interaction effects between variables were studied and incorporated into the model construction. ROC curves and nomograms were generated for visualization. Calibration analysis was performed, and the corresponding net benefit was calculated to adjust the predictive model.

**Results:**

Among the 148 patients, 58 (39.19%) experienced pneumothorax, with a mean age of 62.5 (55.25, 70) years. Multivariate analysis showed that the angle between the puncture needle and the pleura < 50° (*P* = 0.002, *OR*: 3.908, *CI*: 1.621–9.422), preoperative CT suggesting emphysema (*P* = 0.002, *OR*: 3.798, *CI*: 1.600–9.016), atelectasis (*P* = 0.009, *OR*: 3.156, *CI*: 1.331–7.481), and lesion located in the left lung fissure (*P* = 0.008, *OR*: 4.675, *CI*: 14.683) were independent risk factors for pneumothorax after particle implantation in the treatment of advanced lung cancer. Preoperative CT suggesting lesions in the left lung fissure or suggesting emphysema had a significant impact in the nomogram, with probabilities of pneumothorax occurrence at 40% and 38%, respectively. The predictive AUC for the above four risk factors for pneumothorax after particle implantation in the treatment of lung adenocarcinoma was 0.837 (*95% CI*: 0.767–0.908). When the Youden index was 0.59, the sensitivity was 85.56%, specificity was 74.13%, accuracy was 81.01%, positive predictive value was 83.69%, and negative predictive value was 76.78%.

**Conclusion:**

The angle between the puncture needle and the pleura < 50°, preoperative CT suggesting emphysema, atelectasis, and lesion located in the left lung fissure are independent risk factors for pneumothorax after particle implantation in the treatment of advanced lung cancer. Preoperative planning of the puncture path should avoid lung bullae, interlobar fissures, areas of severe emphysema, and atelectasis.

## Introduction

Lung cancer has the highest incidence and mortality rates globally. Approximately 2.5 million people are diagnosed with lung cancer each year, with over 1.6 million deaths attributed to the disease annually (1). The situation of lung cancer in China is particularly severe, with a reported incidence rate of 0.51‰ and a mortality rate of 0.41‰ in 2018 (2). Most lung cancer patients are diagnosed at an advanced stage where surgical resection is not feasible (3). These patients often have limited benefits from traditional chemotherapy and radiation therapy (4). Some advanced lung cancer patients exhibit poor health conditions and are no longer tolerant to conventional treatments (5). Therefore, alternative treatments with fewer side effects or higher tolerability have emerged, including local ablation therapy (6) and radioactive ^125^I particle implantation (7).

In the late 1980s, with the successful development of new radioactive ^125^I particles (8) and the introduction of precise positioning systems guided by ultrasound and CT, as well as computer-based three-dimensional treatment planning systems, the use of close-range radiotherapy with radioactive particles for tumor treatment rapidly expanded (9). Compared to traditional external beam radiation therapy, interstitial radiotherapy offers advantages such as fewer complications, minimal trauma, safety, and effectiveness (10). It can effectively kill tumor cells while minimizing damage to normal tissues (11). Due to its precise targeting, low toxicity, high tumor control rates, it has been rapidly adopted in clinical practice.

Pneumothorax is the most common complication during and after close-range radiotherapy for lung cancer (12). Pneumothorax typically occurs due to the entry of air into the pleural cavity following damage to the visceral pleura, which can be caused by the puncture procedure and particle implantation (13). Pneumothorax requiring closed chest drainage occurs when lung compression exceeds 30% or when symptoms are significant, leading to prolonged hospital stays and increased costs (14). Therefore, minimizing the risk of pneumothorax is crucial during close-range radiotherapy for advanced lung cancer patients. Currently, there is a lack of literature on the analysis of risk factors for pneumothorax after particle implantation in the treatment of advanced lung cancer and the establishment of a predictive model. This study aims to retrospectively analyze the relevant risk factors for pneumothorax after particle implantation in the treatment of advanced lung cancer and develop a practical and reliable predictive model based on objective measurements to effectively predict and minimize the occurrence of postoperative pneumothorax.

## Materials and methods

### Patients

**FIGURE 1:**
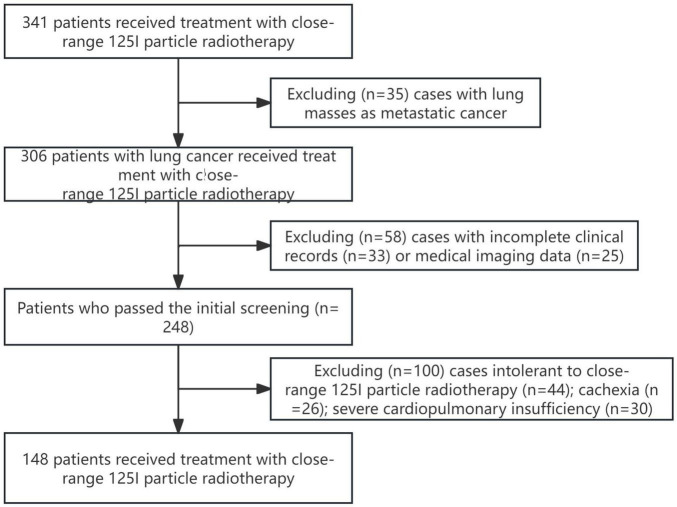
Patient selection flowchart.

This multicenter retrospective cohort study was conducted in accordance with the principles outlined in the Helsinki Declaration. A total of 148 patients who underwent ^125^I particle close-range radiotherapy at *** from December 2022 to December 2023 were included in this study. The study was approved by the Ethics Committee of the *** (Ethics No. YL2021-07), and all patients provided informed consent and signed informed consent forms. Figure 1 illustrates the patient selection process flowchart.

Inclusion criteria: (a) Primary lung cancer; (b) Patients with cardiopulmonary insufficiency or other reasons unable to tolerate surgery and chemotherapy; (c) Patients who refuse surgery and chemotherapy; (d) Patients with postoperative recurrence who are not suitable for further surgery; (e) Failure of external beam radiation therapy, targeted therapy, or immunotherapy; (f) No widespread metastasis; (g) Karnofsky Performance Status (KPS) score ≥ 60 points, expected survival period ≥ 3 months.

Exclusion criteria: Poor image quality; Intolerance to close-range ^125^I radiotherapy; Cachexia; Severe dysfunction of liver, kidney, heart, lung, or brain; Severe anemia, dehydration, and severe disturbances in nutritional metabolism that cannot be corrected or improved in the short term.

### Preoperative preparation

Prior to the procedure, routine examinations including complete blood count, urinalysis, coagulation function tests, infectious disease screening, electrocardiogram, and enhanced chest CT scan were conducted. The scanner used for the procedure was the Discovery VCT PET/CT with 64-slice CT configuration manufactured by GE Healthcare. The implantation gun and pushing needle used were manufactured by Xiangshan in Zhejiang. The puncture needle was the 15–20 cm × 18G needle from Japan’s Hakko. The Treatment Plan System (TPS) for particle therapy was provided by Beijing Feitian Zhaoye Technology Co., Ltd., offering a three-dimensional treatment planning system for radioactive implantation therapy. The radioactive particles (0.8 mm in diameter, 4.5 mm in length, titanium metal fully enclosed shell, particle activity between 18.5–19.6 MBq, half-life of 59.6 days) were produced by Beijing Atom High-Tech Co., Ltd. The particles were sterilized using high-temperature and high-pressure steam sterilization method.

### Intraoperative procedure

Based on the tumor size and location, patients were positioned in supine, lateral, or prone positions for treatment. Preoperatively, 2% lidocaine was used for local anesthesia. CT scans with 5mm thickness of the corresponding tumor layer were performed to measure volume. As per the “Technical Management Standard for Radioactive Particle Implantation Therapy (2017 Edition),” delineation of the target area was done by qualified physicians and physicists. Referring to the 2016 American Brachytherapy Society (ABS) consensus for lung cancer brachytherapy, the recommended prescription dose for ^125^I particle therapy for lung cancer was 80-120Gy (15) when used as a standalone very low dose rate intrathoracic tissue close-range treatment. The Treatment Plan System (TPS) was used to develop the treatment plan. Tumor total volume, planning target volume (PTV), and surrounding critical organs were carefully delineated on each slice. Based on the three orthogonal diameters of the target tumor location and the prescribed matching peripheral dose, the position of the brachytherapy equipment and the number of implanted particles were calculated. Following this, the TPS calculated the dose distribution for the tumor target area and surrounding tissues, generating a dose-volume histogram (DVH). Different positions were selected based on the lesion site, and external positioning grids were placed on the corresponding skin area of the lesion for CT scan localization and needle placement according to the TPS plan. During the procedure, the position and direction of the implanted needle were adjusted based on the specific location of the lung mass on CT scans to ensure the needle entered the lesion depth by passing through the tumor center and being 0.5cm away from the tumor edge. During the surgical procedure, we continuously monitored the patient’s vital signs, including heart rate, blood pressure, and oxygen saturation, while also observing the patient’s consciousness, pain response, and breathing status. After the procedure, the puncture site was compressed for 10–20 min, and the patient’s condition was observed. Postoperatively, the final scan images were verified according to the TPS plan to confirm the position and intensity of the ^125^I particles. Technical success was defined as the postoperative verification of the target area dose reaching the preoperative plan. The quality assessment criteria for particle implantation used the Particle Implant Quality Assessment Criteria of the British Columbia Cancer Research Center, classifying the immediate postoperative verification of target area D90 and V100 as excellent, good, fair, or poor. If the lesion showed inadequate radiation, the process was repeated, and additional ^125^I particles were implanted. Prompt and 24-h CT scans were performed postoperatively to detect and treat any potential postoperative complications. In cases of a small pneumothorax with no symptoms, observation could continue. If lung compression exceeded 30% and the patient experienced chest tightness and dyspnea, closed chest drainage was necessary.

### Data collection

All perioperative inpatient and follow-up data were retrieved from the medical records system. Baseline patient data were collected, including gender, age, smoking history, primary tumor pathology type, preoperative TNM staging, preoperative grading, presence of extrapulmonary metastases, clinical symptoms (cough, sputum production, chest tightness, dyspnea, wheezing), treatment history (chemotherapy, radiotherapy). Parameters related to the close-range radiotherapy with particle implantation were assessed, such as whether the pleural puncture angle was less than 50°, preoperative CT findings (pulmonary emphysema, obstructive pneumonia, lung collapse, superior vena cava obstruction sign, location within interlobar fissures), preoperative long diameter, and lesion location.

### Data analysis

All statistical analyses were performed using R software (version 4.2.1). For numerical variables, normality tests were first conducted. When the data did not follow a normal distribution, the corresponding variables were reported with their median and interquartile range; for data that did not meet the normality assumption, the Wilcoxon test was used to compare the differences between two groups. For categorical variables, the chi-square test was employed for group comparisons only when the theoretical frequency was greater than 5 and the total sample size was ≥ 40. After data cleaning, a single-factor binary logistic regression analysis was performed using the glm function to identify important variables. Subsequently, a multiple-factor binary logistic regression analysis was conducted using the rms (version 6.4.0) and Resource Selection (version 0.3–5) packages. The variable selection strategy was to include only those variables with a p-value less than 0.05 in the single-factor analysis into the multiple-factor model. For data visualization, the ggplot2 package was used to create forest plots, and the pROC package was employed for ROC analysis, with the analysis results visualized using ggplot2. The pROC package automatically adjusts the order of the results to ensure that the ROC curve appears concave. Finally, calibration analyses were performed on the binary logistic regression model built after data cleaning, with visualizations completed using the rms package (version 6.4.0) and Resource Selection package (version 0.3–5). Additionally, the rmda package (version 1.6) was used in conjunction with ggplot2 (version 3.3.6) to calculate the corresponding net benefits and visualize the results.

## Results

### Baseline characteristics

A total of 148 patients who underwent ^125^I particle close-range radiotherapy at the *** from December 2022 to December 2023 were included in this study. Table 1 summarizes the baseline clinical characteristics of the patients based on the occurrence of pneumothorax after ^125^I particle close-range radiotherapy. Out of the 148 patients, 58 cases (39.19%) experienced pneumothorax, with a mean age of 62.5 (55.25, 70) years. Among them, there were 30 smokers (20.3%). The primary tumor pathology types included 16 cases of lung adenocarcinoma (10.8%), 22 cases of lung squamous cell carcinoma (14.9%), 1 case of neuroendocrine carcinoma (0.7%), and 19 cases of small cell lung cancer (12.8%). Preoperative grading showed 12 cases at stage III (8.1%) and 46 cases at stage IV (31.1%). Among the patients who experienced pneumothorax, preoperative examinations indicated extrapulmonary metastases in 19 cases (12.8%). Preoperative clinical symptoms included cough and sputum production in 25 cases each (16.9%), chest tightness and dyspnea in 30 cases each (20.3%). Twenty-two patients (14.9%) had undergone radiotherapy before the procedure, and 18 patients (12.2%) had received chemotherapy prior to the treatment.

**TABLE 1:** Baseline clinical characteristics of patients with and without pneumothorax.

Characteristics	Pneumothorax	Non-pneumothorax	*P*-value
**n**	**58**	**90**	
Gender, *n* (%)			0.639
Male	30 (20.3%)	43 (29.1%)	
Female	28 (18.9%)	47 (31.8%)	
Age, median (IQR)	62.5 (55.25, 70)	62 (56, 70)	0.987
Smoking, *n* (%)			0.838
Yes	30 (20.3%)	45 (30.4%)	
No	28 (18.9%)	45 (30.4%)	
Primary tumor pathology type, *n* (%)			0.082
Lung adenocarcinoma	16 (10.8%)	40 (27%)	
Lung squamous cell carcinoma	22 (14.9%)	22 (14.9%)	
Neuroendocrine carcinoma	1 (0.7%)	0 (0%)	
Cancer with SMARCA4 deficiency	0 (0%)	1 (0.7%)	
Small cell lung cancer	19 (12.8%)	27 (18.2%)	
Preoperative, T, *n* (%)			0.932
2	19 (12.8%)	31 (20.9%)	
3	25 (16.9%)	36 (24.3%)	
4	14 (9.5%)	23 (15.5%)	
Preoperative, N, *n* (%)			0.190
1	7 (4.7%)	4 (2.7%)	
2	24 (16.2%)	36 (24.3%)	
3	27 (18.2%)	50 (33.8%)	
Preoperative, M, *n* (%)			0.512
0	17 (11.5%)	22 (14.9%)	
1	41 (27.7%)	68 (45.9%)	
Preoperative, grade, *n* (%)			0.596
3	12 (8.1%)	22 (14.9%)	
4	46 (31.1%)	68 (45.9%)	
Extrapulmonary metastasis, *n* (%)			0.832
Yes	19 (12.8%)	31 (20.9%)	
No	39 (26.4%)	59 (39.9%)	
Cough, *n* (%)			0.978
Yes	25 (16.9%)	39 (26.4%)	
No	33 (22.3%)	51 (34.5%)	
Coughing up phlegm, *n* (%)			0.810
Yes	25 (16.9%)	37 (25%)	
No	33 (22.3%)	53 (35.8%)	
Shortness of breath, *n* (%)			0.639
Yes	30 (20.3%)	43 (29.1%)	
No	28 (18.9%)	47 (31.8%)	
Asthma, *n* (%)			0.463
Yes	30 (20.3%)	41 (27.7%)	
No	28 (18.9%)	49 (33.1%)	
Radiotherapy, *n* (%)			0.392
Yes	22 (14.9%)	28 (18.9%)	
No	36 (24.3%)	62 (41.9%)	
Chemotherapy, *n* (%)			0.171
Yes	18 (12.2%)	38 (25.7%)	
No	40 (27%)	52 (35.1%)	

Data are presented as *n* (%) and quartiles.

The differences between the non-pneumothorax group and pneumothorax group in terms of the angle between the puncture needle and the pleura being < 50°, preoperative CT findings indicating pulmonary emphysema, obstructive pneumonia, lung collapse, superior vena cava obstruction, and location within the interlobar fissures were statistically significant (all *P* < 0.05), as shown in Table 2. However, there were no statistically significant differences in the number of tumors, preoperative long diameter, lung lobe, and lung segment aspects (all *P* > 0.05).

**TABLE 2:** Descriptive analysis of factors associated with pneumothorax and non-pneumothorax patients.

Characteristics	Pneumothorax	Non-pneumothorax	*P*-value
**n**	**58**	**90**	
The angle between the puncture needle and the pleura is less than 50 degrees, *n* (%)			< 0.001
Yes	41 (27.7%)	31 (20.9%)	
No	17 (11.5%)	59 (39.9%)	
Pulmonary emphysema, *n* (%)			<0.001
Yes	41 (27.7%)	25 (16.9%)	
No	17 (11.5%)	65 (43.9%)	
Obstructive pneumonia, *n* (%)			0.004
Yes	32 (21.6%)	28 (18.9%)	
No	26 (17.6%)	62 (41.9%)	
Interlobar fissure, *n* (%)			0.002
Not located in the interlobar fissure	31 (20.9%)	22 (14.9%)	
Right pulmonary oblique fissure	9 (6.1%)	17 (11.5%)	
Left pulmonary oblique fissure	9 (6.1%)	33 (22.3%)	
Right pulmonary horizontal fissure	9 (6.1%)	18 (12.2%)	
Preoperative long diameter, median (IQR)	4.4 (3.5, 5.2)	4.4 (3.525, 5.15)	0.898
Pulmonary lobe, *n* (%)			0.570
right	18 (12.2%)	32 (21.6%)	
left	40 (27%)	58 (39.2%)	
Pulmonary segment, *n* (%)			0.303
Upper lobe	16 (10.8%)	36 (24.3%)	
Middle lobe	6 (4.1%)	8 (5.4%)	
Lower lobe	36 (24.3%)	46 (31.1%)	
Atelectasis, *n* (%)			<0.001
Yes	39 (26.4%)	29 (19.6%)	
No	19 (12.8%)	61 (41.2%)	
Superior vena cava obstruction, *n* (%)			0.023
Yes	19 (12.8%)	15 (10.1%)	
No	39 (26.4%)	75 (50.7%)	

Data are presented as *n* (%) and quartiles.

### Factors affecting postoperative pneumothorax in lung adenocarcinoma patients undergoing particle implantation close-range radiotherapy

**FIGURE 2:**
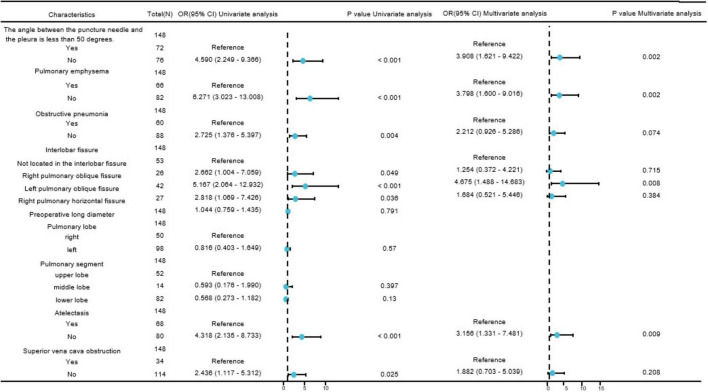
Forest plot of risk factors affecting postoperative pneumothorax in lung adenocarcinoma patients undergoing particle implantation close-range radiotherapy. Visualizing complex tables displaying statistical effect sizes and confidence intervals.

In the single-factor analysis, differences were found to be statistically significant (all *P* < 0.05) in the angle between the puncture needle and the pleura being < 50°(*P <* 0.001), preoperative CT findings indicating pulmonary emphysema (*P <* 0.001), obstructive pneumonia (*P* = 0.004), right pulmonary oblique fissure (*P* = 0.049), left pulmonary oblique fissure (*P <* 0.001), right pulmonary horizontal fissure (*P* = 0.036), atelectasis (*P <* 0.001), and superior vena cava obstruction (*P* = 0.025). The multi-factor analysis revealed that among these factors, the angle between the puncture needle and the pleura being < 50° (*P* = 0.002, *OR*: 3.908, *CI*: 1.621–9.422), preoperative CT findings indicating pulmonary emphysema (*P* = 0.002, *OR*: 3.798, *CI*: 1.600–9.016), lung collapse (*P* = 0.009, *OR*: 3.156, *CI*: 1.331–7.481), and the lesion being located in the left lung oblique fissure (*P* = 0.008, *OR*: 4.675, *CI*: 1.488–14.683) were identified as independent risk factors for postoperative pneumothorax in advanced lung cancer patients undergoing particle implantation close-range radiotherapy. More details can be found in Table 3 and Figure 2.

**TABLE 3:** Risk factor analysis for postoperative pneumothorax after particle implantation in the treatment of lung adenocarcinoma.

Characteristics	Total (N)	Univariate analysis		Multivariate analysis	
		**Odds Ratio (*95% CI*)**	***P*-value**	**Odds Ratio (*95% CI*)**	***P*-value**
The angle between the puncture needle and the pleura is less than 50 degrees	148				
Yes	72	Reference		Reference	
No	76	4.590 (2.249–9.366)	<0.001	3.908 (1.621–9.422)	0.002
Pulmonary emphysema	148				
Yes	66	Reference		Reference	
No	82	6.271 (3.023–13.008)	<0.001	3.798 (1.600–9.016)	0.002
Obstructive pneumonia	148				
Yes	60	Reference		Reference	
No	88	2.725 (1.376–5.397)	0.004	2.212 (0.926–5.286)	0.074
Interlobar fissure	148				
Not located in the interlobar fissure	53	Reference		Reference	
Right pulmonary oblique fissure	26	2.662 (1.004–7.059)	0.049	1.254 (0.372–4.221)	0.715
Left pulmonary oblique fissure	42	5.167 (2.064–12.932)	<0.001	4.675 (1.488–14.683)	0.008
Right pulmonary horizontal fissure	27	2.818 (1.069–7.426)	0.036	1.684 (0.521–5.446)	0.384
Preoperative long diameter, median (IQR)	148	1.044 (0.759–1.435)	0.791		
Pulmonary lobe	148				
right	50	Reference			
left	98	0.816 (0.403–1.649)	0.570		
Pulmonary segment	148				
upper lobe	52	Reference			
middle lobe	14	0.593 (0.176–1.990)	0.397		
lower lobe	82	0.568 (0.273–1.182)	0.130		
Atelectasis	148				
Yes	68	Reference		Reference	
No	80	4.318 (2.135–8.733)	<0.001	3.156 (1.331–7.481)	0.009
Superior vena cava obstruction	148				
Yes	34	Reference		Reference	
No	114	2.436 (1.117–5.312)	0.025	1.882 (0.703–5.039)	0.208

### Construction of a nomogram

**FIGURE 3:**
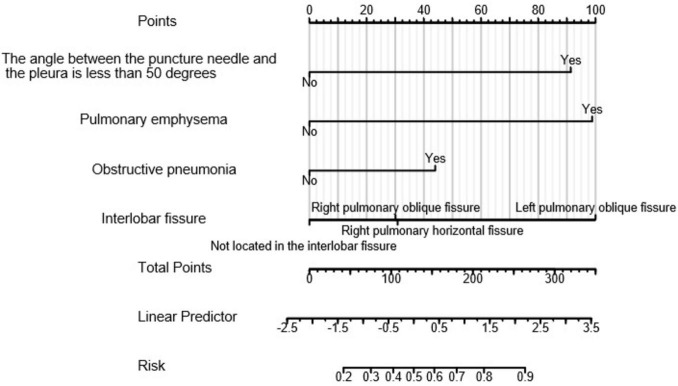
Clinical prediction model for postoperative pneumothorax in lung adenocarcinoma patients undergoing particle implantation close-range radiotherapy. Points: Represents the individual score corresponding to each predictive variable at different values. Variable: Indicates the values of each variable in the model and their corresponding scores. Total Points: Represents the total score obtained by summing the individual scores corresponding to all variable values. Linear Predictor: Indicates the linear predicted value. Right Scale: Represents the scale and range of values for the left-side title text.

A nomogram (as shown in Figure 3) was constructed based on the results selected from the multiple-factor logistic regression analysis. The score for each independent predictive factor was plotted and summed continuously to obtain the total score. In predicting the occurrence of postoperative pneumothorax in advanced lung cancer patients undergoing particle implantation close-range radiotherapy, the factors of preoperative CT findings indicating the lesion located in the left lung oblique fissure or indicating pulmonary emphysema had a significant impact on the nomogram, with probabilities of pneumothorax occurrence of 40% and 38%, respectively.

### Evaluation of the nomogram

**FIGURE 4:**
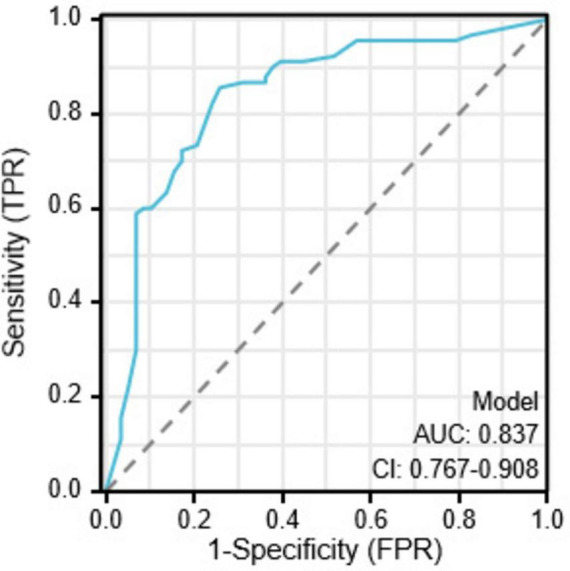
Combined index ROC curve for postoperative pneumothorax in advanced lung cancer patients undergoing particle implantation close-range radiotherapy.

**FIGURE 5:**
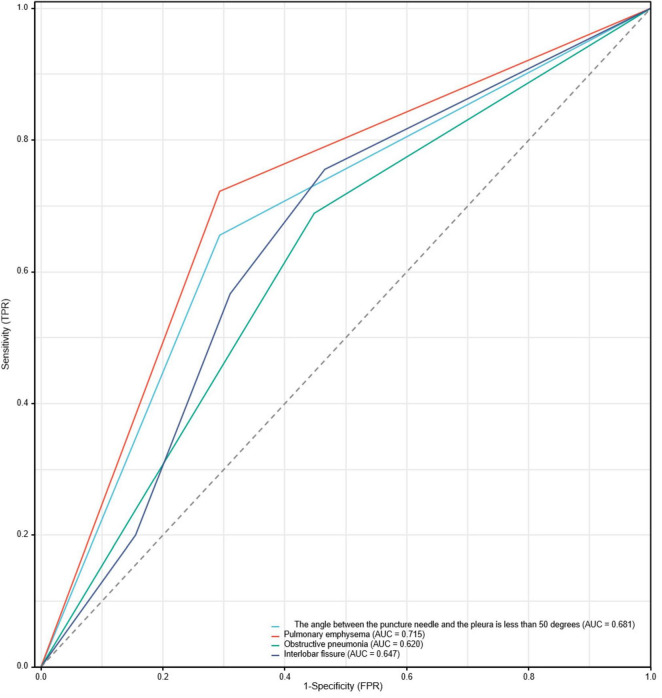
ROC curve for postoperative pneumothorax in advanced lung cancer patients undergoing particle implantation close-range radiotherapy.

The ROC curve analysis results indicate that four risk factors - the angle between the puncture needle and the pleura being < 50°, preoperative CT suggesting pulmonary emphysema, atelectasis, and the lesion located in the left lung oblique fissure - have an AUC of 0.837 (*95% CI*: 0.767–0.908) for predicting postoperative pneumothorax in advanced lung cancer patients undergoing particle implantation close-range radiotherapy. The sensitivity is 85.56%, specificity is 74.13%, accuracy is 81.01%, positive predictive value is 83.69%, and negative predictive value is 76.78% when the Youden index is 0.59, as shown in Figure 4. The ROC curve results for the angle between the puncture needle and the pleura being < 50°, pulmonary emphysema, obstructive pneumonia, and whether it is located in the interlobar fissure are shown in Figure 5, with specific values provided in Tables 3, 4. Preoperative CT suggesting pulmonary emphysema, obstructive pneumonia, lesion location in the interlobar fissure, and the angle between the puncture needle and the pleura being < 50° show good diagnostic performance in predicting the outcome as shown in Table 5.

**TABLE 4:** Diagnostic ROC curve AUC results table.

Predictive variables	Area under the curve (*AUC*)	Confidence interval (*CI*)
The angle between the puncture needle and the pleura is less than 50 degrees	0.681	0.604–0.758
Pulmonary emphysema	0.715	0.639–0.790
Obstructive pneumonia	0.620	0.540–0.701
Interlobar fissure	0.647	0.556–0.737

When AUC > 0.5, the closer AUC is to 1, the better the variable is at predicting the outcome. AUC values between 0.5 and 0.7 indicate low accuracy, between 0.7 and 0.9 indicate moderate accuracy, and above 0.9 indicate high accuracy. AUC = 0.5 indicates that the variable has no predictive value and is not useful for diagnosis.

**TABLE 5:** ROC information table.

Predictive variables	Sensitivity	Specificity	Accuracy	Positive predictive value	Negative predictive value	Youden index
The angle between the puncture needle and the pleura is less than 50 degrees	65.55%	70.69%	67.57%	77.63%	56.94%	0.36
Pulmonary emphysema	72.22%	70.69%	71.62%	79.27%	62.12%	0.42
Obstructive pneumonia	68.89%	55.17%	63.51%	70.45%	53.33%	0.24
Interlobar fissure	75.55%	53.44%	66.89%	71.57%	58.49%	0.29

Partial ROC-related information and data for each predictive variable at their respective optimal cut-off values. Youden Index = Sensitivity ++ Specificity−1.

**FIGURE 6:**
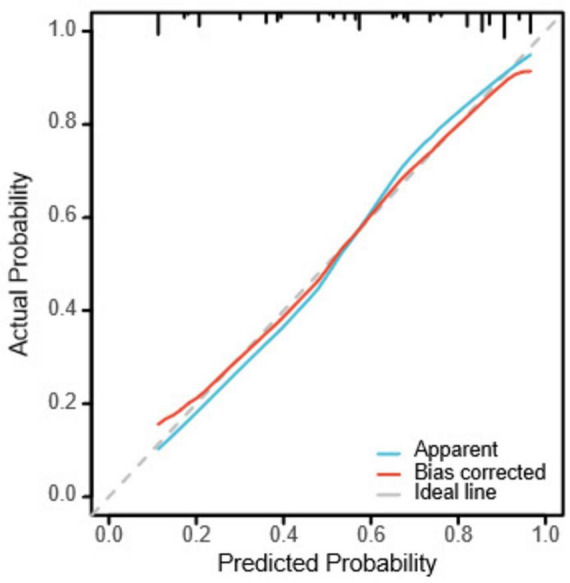
Calibration curve for postoperative pneumothorax after particle implantation for the treatment of lung adenocarcinoma at a short distance. The x-axis represents the model-predicted survival probability, while the y-axis represents the observed survival probability. The Apparent curve represents the predicted curve, the Bias-corrected curve represents the calibration curve, and the Ideal curve represents the ideal curve; closer alignment to the diagonal line indicates better fit. The distribution plot on the top axis shows the distribution of predicted probabilities, with denser areas indicating more samples at that probability.

**FIGURE 7:**
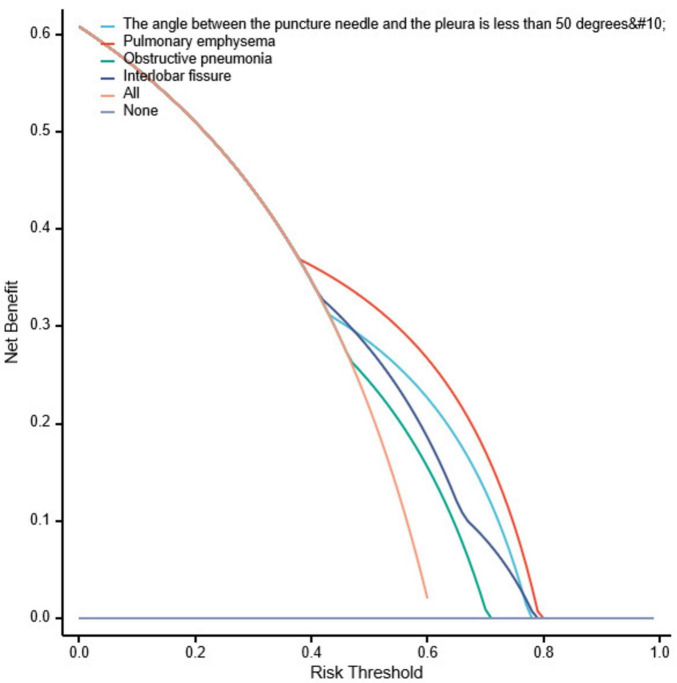
Decision curve analysis for postoperative pneumothorax after particle implantation for the treatment of lung adenocarcinoma at a short distance. Decision curve analysis (DCA) is used to describe the change in net benefit values as the risk probability threshold varies when interventions are made based on model predictions. The x-axis represents the risk probability threshold, and the y-axis represents the net benefit (the difference between the benefit of intervening for true positive patients at this high-risk probability threshold and the cost of intervening for false positive patients). Each curve represents the change in net benefit as the high-risk probability threshold varies for each model, where “All” represents intervention for the entire population, and “None” represents no intervention, resulting in a net benefit of 0. Models whose curves are close to the two reference lines (All and None) are considered to have low utility, while those above the reference lines in a significant probability threshold range are viewed as better models.

Bootstrapping was used to resample the original dataset 1000 times to establish a simulated dataset. The calibration curve (Figure 6) indicates good consistency between the discriminative ability of the predictive model and the actual discrimination of postoperative pneumothorax in advanced lung cancer patients undergoing particle implantation close-range radiotherapy. The likelihood ratio chi-square value is 53.187, with *P* < 0.01, indicating that at least one variable in the fitted model has a statistically significant odds ratio, meaning the model is overall meaningful. The discriminative ability of the model is evaluated using the C-index, which is 0.837 (0.766–0.908), indicating moderate accuracy. The calibration assessment is done using the Hosmer-Lemeshow Goodness of Fit test, with a chi-square value of 15.762 and *P* = 0.459, indicating no significant difference between predicted and observed values, suggesting good model fit. Decision Curve Analysis (DCA) demonstrates the effectiveness of the two nomograms across a wide range of probability thresholds (Figure 7). The intercept for the pulmonary emphysema model is –0.4947, AIC value is 175.280, indicating the best model fit, with P < 0.01.

### Severity and treatment of pneumothorax

**FIGURE 8:**
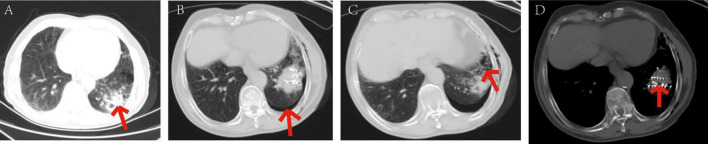
Patients with mild pneumothorax did not receive any specific treatment. The patient, an elderly female, diagnosed with adenocarcinoma of the left lower lobe of the lung. Under our department’s care, underwent CT-guided percutaneous ^125^I particle implantation close-range radiotherapy. (Indicated by the red arrow) **(a)** preoperative CT lung window showing a lesion measuring 5.6 × 6.2 cm. **(b)** Immediate postoperative CT reexamination showing mild pneumothorax formation in the lung window. **(c)** Intraoperative CT lung window showing pulmonary emphysema adjacent to the lesion in the left lower lobe and cavities visible within the lesion. **(d)** Intraoperative mediastinal window showing the implantation of 91 particles in the lesion of the left lower lobe. The patient’s mild pneumothorax was self-absorbed, and on reexamination 2 days later, the pneumothorax had completely disappeared.

**FIGURE 9:**
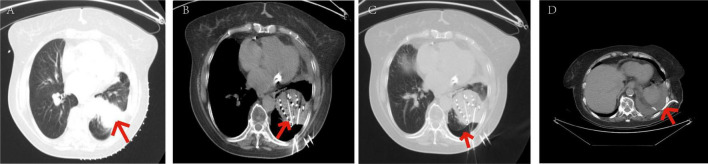
Symptoms relieved in patients with moderate pneumothorax through closed chest tube drainage. The patient, an elderly male, diagnosed with adenocarcinoma of the left lower lobe of the lung. Under our department’s care, underwent percutaneous ^125^I particle implantation close-range radiotherapy. (Indicated by the red arrow) **(a)** preoperative CT lung window showing a lesion measuring 7.3 × 4.9 cm. **(b)** Intraoperative mediastinal window showing the implantation of 109 particles in the lesion of the left lower lobe. **(c)** Intraoperative CT lung window showing the formation of moderate pneumothorax. **(d)** Postoperative closed chest tube drainage placement and drainage procedure, with approximately 1000 ml of gas drained over 3 days, leading to significant improvement in symptoms. On the 4th day, the closed chest tube drainage was removed, with a small amount of pleural effusion in the left lower lobe.

Among 58 cases of pneumothorax, 36 cases (62.06%) were diagnosed as mild pneumothorax. The incidence rates of

moderate and severe pneumothorax were 13 cases (36.11%) and 9 cases (25%), respectively. Patients with mild pneumothorax did not receive any specific treatment, as shown in Figure 8. Among the 13 patients with moderate pneumothorax, 6 received conservative treatment, and 4 showed improvement postoperatively, transitioning to mild pneumothorax. Patients with moderate and severe pneumothorax (7 and 9 cases, respectively) had their symptoms relieved through closed chest tube drainage, with symptoms resolving within a maximum of 6 days, as shown in Figure 9.

## Discussion

Lung cancer is the most common malignant tumor in China (15). In the late stages of the tumor, when the opportunity for surgical resection is lost, clinical practice often involves combined chemotherapy, palliative radiotherapy, comprehensive treatment, and other approaches (16). Traditional external radiotherapy has many drawbacks. When the irradiation area exceeds 120cm^2^ and the dose exceeds 40Gy, radiation pneumonitis is more likely to occur (17). Due to the limitations of normal lung tissue in tolerating radiation doses, traditional external radiotherapy cannot achieve effective control doses for lung cancer. In recent years, minimally invasive treatments have become one of the alternative treatment methods for inoperable lung cancer, including particle implantation (5) and ablation (18), among others. These methods can directly or indirectly damage tumor cells and play a significant role in the treatment of tumors that are not amenable to surgical resection. Particle therapy involves emitting continuous, short-range radiation from a radiation source, causing maximum damage to tumor tissue while sparing surrounding normal tissues from radiation injury, thereby achieving the treatment goal. When treating lung cancer with ^125^I particle implantation, the radiation resistance of hypoxic cells is reduced, and under conditions of sustained low-dose irradiation, hypoxic cells can reoxygenate, leading to maximum destructive damage to tumor cells (19). The close-range radiotherapy technique of permanently implanting ^125^I particles between tissues can effectively control the local development of tumors, reduce in-situ tumor recurrence, and significantly improve the survival period of tumor patients.

Pneumothorax is the most common complication during and after close-range radiotherapy for lung cancer (20). It can manifest as varying degrees of chest tightness, shortness of breath, and respiratory distress. Severe cases can lead to mediastinal shift and lung collapse within a short period, causing cardiopulmonary dysfunction. During close-range radiotherapy, both the puncture itself and particle implantation can damage the visceral pleura, leading to pneumothorax. The reported incidence of pneumothorax after lung biopsy in the past generally ranges from 2.4% to 60% (with an average incidence of 20%) (21), while the probability of pneumothorax after late-stage lung cancer close-range radiotherapy has not been clearly reported. In this study, among 148 patients with late-stage lung cancer treated with particle implantation close-range radiotherapy, 58 patients (39.19%) experienced pneumothorax, with an average age of 62.5 (55.25, 70) years. Among them, there were 30 smokers (20.3%). Clinical symptoms before surgery included cough and sputum in 25 patients each (16.9%) and chest tightness, shortness of breath, and wheezing in 30 patients each (20.3%). Twenty-two patients (14.9%) received radiotherapy before surgery, and 18 patients (12.2%) received chemotherapy before surgery. The incidence of pneumothorax after close-range radiotherapy for late-stage lung cancer is slightly higher than that after lung biopsy, possibly due to the damage to the visceral pleura caused by particle implantation and an increase in the number of needle insertions.

In this study cohort, there were statistically significant differences in the characteristics between the group without pneumothorax and the group with pneumothorax, including whether the angle between the puncture needle and the pleura was < 50°, preoperative CT findings of pulmonary emphysema, obstructive pneumonia, lung collapse, superior vena cava obstruction, and whether the lesion was located at the interlobar fissure (all with *P* < 0.05). Univariate analysis showed statistically significant differences in the angle between the puncture needle and the pleura being < 50°, preoperative CT findings of pulmonary emphysema, obstructive pneumonia, whether the lesion was located at the interlobar fissure, lung collapse, and superior vena cava obstruction (all with *P* < 0.05). Further multivariate analysis revealed that the angle between the puncture needle and the pleura being < 50° (*P* = 0.002, *OR*: 3.908, *CI*: 1.621–9.422), preoperative CT findings of pulmonary emphysema (*P* = 0.002, *OR*: 3.798, *CI*: 1.600–9.016), lung collapse (*P* = 0.009, *OR*: 3.156, *CI*: 1.331–7.481), and the lesion being located at the left lung fissure (*P* = 0.008, *OR*: 4.675, *CI*: 14.683) were independent risk factors for pneumothorax after particle implantation close-range radiotherapy for late-stage lung cancer. This is consistent with the conclusion that pulmonary emphysema is closely related to an increased risk of pneumothorax, as found by Tyler Sargent. The angle between the puncture needle and the pleura is an independent factor affecting the incidence of pneumothorax, consistent with the study by Jane P. Ko et al., which showed that an angle < 80°, especially < 50°, is significantly associated with pneumothorax incidence, and there is a clear negative correlation between the needle insertion angle and pneumothorax incidence (22). The explanation for this finding may be that needle entry at a shallow angle into the pleura creates a narrow and sharp puncture hole that is more likely to be elongated by traction. We may be the first to discover that lesions located at the left lung fissure are more prone to pneumothorax after particle implantation close-range radiotherapy. While no previous conclusions have been drawn, it can be hypothesized that compared to the right lung, the left lung has a larger air volume, allowing for more available space for needle guidance under imaging, which can be adjusted to avoid potential vessels and pulmonary emphysema on the images (23). Additionally, the anatomical structure of the bronchial tree (i.e., the branching angles in the left upper lobe, apical posterior, and anterior segments relative to the lingular lobe at larger angles) may also provide a reasonable explanation (24).

Based on the results of the multivariable logistic regression analysis, a Nomogram prediction model was constructed to help identify patients undergoing close-range radiotherapy who are at risk of developing postoperative pneumothorax. This model allows for risk stratification and provides information for preoperative interventions or intraoperative monitoring. In predicting the occurrence of pneumothorax after particle implantation close-range radiotherapy for lung adenocarcinoma, preoperative CT findings suggesting the lesion is located at the left lung fissure or indicating pulmonary emphysema have a significant impact in the Nomogram, with probabilities of pneumothorax occurrence being 40% and 38%, respectively. The four risk factors of the angle between the puncture needle and the pleura being < 50°, preoperative CT findings of pulmonary emphysema, lung collapse, and the lesion being located at the left lung fissure have an AUC of 0.837 (*95% CI*: 0.767–0.908) for predicting pneumothorax after particle implantation close-range radiotherapy for lung adenocarcinoma. At a Youden index of 0.59, the sensitivity is 85.56%, specificity is 74.13%, accuracy is 81.01%, positive predictive value is 83.69%, and negative predictive value is 76.78%. Preoperative CT findings suggesting pulmonary emphysema have an AUC of 0.715, with a Youden index of 0.42, sensitivity of 72.22%, specificity of 70.69%, accuracy of 71.62%, positive predictive value of 79.27%, and negative predictive value of 62.12%. The Nomogram model shows good diagnostic performance for predicting outcomes related to whether the lesion is located at the interlobar fissure, with an AUC of 0.647, Youden index of 0.29, sensitivity of 75.55%, specificity of 53.44%, accuracy of 66.89%, positive predictive value of 71.57%, and negative predictive value of 58.49%. Bootstrapping was used to resample the original dataset 1000 times to establish a simulated dataset. The calibration curve indicates good consistency between the predictive model and the actual occurrence of pneumothorax after particle implantation close-range radiotherapy for lung adenocarcinoma. The likelihood ratio chi-square value of 53.187 (*P* < 0.01) suggests that the fitted model is overall significant. The discriminative ability of the model is evaluated using the C-index, which is 0.837 (0.766–0.908), indicating moderate accuracy. Decision curve analysis (DCA) demonstrates the effectiveness of the Nomogram models across a wide range of threshold probability values. The pulmonary emphysema model has a constant/intercept of –0.4947, an AIC value of 175.280, and the best model fit with *P* < 0.01.

Pneumothorax is more likely to occur in patients where the angle between the puncture needle and the pleura is < 50°, preoperative CT indicates pulmonary emphysema, lung collapse, or the lesion is located at the left lung fissure. To reduce the occurrence of postoperative pneumothorax, preoperative planning of the puncture path should aim to avoid lung bullae, interlobar fissures, and areas of severe pulmonary emphysema. There are several studies on preventing pneumothorax after lung biopsy procedures: Geoffrey Bourgeais et al. suggest that saline irrigation during percutaneous biopsy under CT guidance with subsequent coaxial system removal significantly reduces the rate of pneumothorax occurrence and decreases the need for chest tube placement (25). Lynn Leng et al. (26) propose the rapid flip maneuver, where the patient is repositioned with the biopsy side down after needle removal to reduce alveolar size around the needle tract, leading to airway closure and a decrease in the pleural pressure gradient, thereby preventing pneumothorax (26). In high-risk patients identified by the model in this study for pneumothorax during close-range radiotherapy, these approaches can be considered to reduce the incidence of pneumothorax.

## Conclusion

In summary, the pneumothorax risk prediction model demonstrates high accuracy in predicting the likelihood of pneumothorax during particle implantation close-range radiotherapy for advanced lung cancer. It can be applied preoperatively to estimate the risk probability, assess risk values, and prepare appropriate measures in advance to enhance the safety of surgery involving particle implantation close-range radiotherapy for advanced lung cancer. This model holds significant clinical value and practical implications.

## Data Availability

The raw data supporting the conclusions of this article will be made available by the authors, without undue reservation.
